# New estimates of the number of children living with substance misusing parents: results from UK national household surveys

**DOI:** 10.1186/1471-2458-9-377

**Published:** 2009-10-08

**Authors:** Victoria Manning, David W Best, Nathan Faulkner, Emily Titherington

**Affiliations:** 1 National Addiction Centre/Institute of Psychiatry/South London and Maudsley NHS Trust, 1-4 Windsor Walk, Denmark Hill, London, SE5 8AF, UK; 2 Criminal Justice Research Centre, Room 822A, University of the West of Scotland, Hamilton Campus, Almada Street, Hamilton, Lanarkshire ML3 0JB, UK

## Abstract

**Background:**

The existing estimates of there being 250,000 - 350,000 children of problem drug users in the UK (ACMD, 2003) and 780,000 - 1.3 million children of adults with an alcohol problem (AHRSE, 2004) are extrapolations of treatment data alone or estimates from other countries, hence updated, local and broader estimates are needed.

**Methods:**

The current work identifies profiles where the risk of harm to children could be increased by patterns of parental substance use and generates new estimates following secondary analysis of five UK national household surveys.

**Results:**

The Health Survey for England (HSfE) and General Household Survey (GHS) (both 2004) generated consistent estimates - around 30% of children under-16 years (3.3 - 3.5 million) in the UK lived with at least one binge drinking parent, 8% with at least two binge drinkers and 4% with a lone (binge drinking) parent. The National Psychiatric Morbidity Survey (NPMS) indicated that in 2000, 22% (2.6 million) lived with a hazardous drinker and 6% (705,000) with a dependent drinker. The British Crime Survey (2004) and NPMS (2000) indicated that 8% (up to 978,000) of children lived with an adult who had used illicit drugs within that year, 2% (up to 256,000) with a class A drug user and 7% (up to 873,000) with a class C drug user. Around 335,000 children lived with a drug dependent user, 72,000 with an injecting drug user, 72,000 with a drug user in treatment and 108,000 with an adult who had overdosed. Elevated or cumulative risk of harm may have existed for the 3.6% (around 430,000) children in the UK who lived with a problem drinker who also used drugs and 4% (half a million) where problem drinking co-existed with mental health problems. Stronger indicators of harm emerged from the Scottish Crime Survey (2000), according to which 1% of children (around 12,000 children) had witnessed force being used against an adult in the household by their partner whilst drinking alcohol and 0.6% (almost 6000 children) whilst using drugs.

**Conclusion:**

Whilst harm from parental substance use is not inevitable, the number of children living with substance misusing parents exceeds earlier estimates. Widespread patterns of binge drinking and recreational drug use may expose children to sub-optimal care and substance-using role models. Implications for policy, practice and research are discussed.

## Background

Child protection cases that feature in the UK media are reminders of how babies and children can be vulnerable to harm from parents and other adults and how frequently these cases involve binge or chronic substance use. According to ACMDs Hidden Harm report (2003) [1], parental drug use can compromise a child's health and development from conception onwards. Parental substance misuse has been associated with genetic, developmental, psychological, psychosocial, physical, environmental and social harms to children [1-5]. The unborn child may be adversely affected by direct exposure to alcohol and drugs through maternal substance use [6-8]. The risk of harm however, depends on the age of the child, the nature and patterns of substance use and contextual factors in which the substance use occurs [9]. Social deprivation and the financing of drug or alcohol consumption may restrict money allocated to meet basics needs for the child. Inadequate monitoring, early exposure to substance taking behaviours, modelling behaviour and the failure to provide a nurturing environment can result in maladaptive and dysfunctional behaviour and other poor outcomes for the child [for reviews see [1,2,10]].

The potential for harm is not likely to be limited to dependent substance use. Binge drinking or regular non-problematic drug use can affect a person's control of emotions, judgement and ability to respond to situations, particularly during periods of intoxication and withdrawal. Being under the influence of substances may affect parental responsiveness to the physical or emotional needs of a child. For example, while parents recover from a hangover, babies and young children may be under-stimulated, whilst older children may carry the burden of household responsibilities and caring roles for siblings [11].

The limited research attempting to unveil the types of harm associated with parental substance misuse is largely restricted to retrospective cohort studies. Much of this work has attempted to identify adverse childhood experiences (ACEs) in the context of parental alcohol misuse among unhealthy/addicted adult populations [12,13]. Exposure to parental alcohol abuse is highly associated with ACEs [14]. Compared to persons reporting no ACEs, the risk of heavy drinking, alcoholism and depression in adulthood is significantly increased by the presence of multiple ACEs [15]. Another study examining ten ACEs (childhood emotional, physical, and sexual abuse, witnessing domestic violence, parental separation or divorce, growing up with drug abusing, alcohol abusing, mentally ill, suicidal, or criminal household members) found that the risk of having all of these was significantly greater among adult respondents who reported parental alcohol abuse [16]. Due to its sensitive nature and parents' fear of social services involvement, it is extremely difficult to conduct research to answer these questions. We are yet to determine the effects parental heavy drinking episodes and recreational illicit drug use have on children.

The latest drug strategy document for England estimates that there are around 330,000 problem drug users in England [17] - the majority of whom are of a parenting age. The document places heavy emphasis on reducing the risk of harm to children of drug-misusers, expressing a commitment to addressing the needs of parents and children by working with whole families to prevent drug use and reduce risk. In terms of the prevention agenda, it aims to promote the sharing of information across institutions e.g. ensure children's social services are aware of drug-using parents where children could be at risk and promises to 'expand their approach so that it increasingly focuses on young children and families before problems have arisen'. Linked to this is a commitment to take a 'wider preventative view' focussing on all substances including alcohol misuse. Regarding treatment the aims are to prioritise cases causing the most harm to families, by ensuring prompt access into effective treatment, assessment of family needs and intensive parenting support. It also aims to ensure that drug-misusing parents become a target group for new parenting experts, with Family Intervention Projects for families considered to be 'at-risk'.

When it comes to estimating the number of children at risk of harm from parental substance misuse, two sources are used as the epidemiological data on which the above targets are centred. The ACMD (2003) report 'Hidden Harm' estimated there being between 250,000 - 350,000 children of problem drug users in the UK, representing 2-3% of all under-16 years olds and the 2004 Alcohol Harm Reduction Strategy for England [18], estimated there being 780, 000 - 1.3 million children living with adults with an alcohol problem. There are however limitations with both of these estimates. The number of children estimated to be living with drug- misusing parents is an extrapolation of treatment data alone, that is, records of drug users presenting for treatment until the end of 2000. There is a concern that women are less likely to access treatment [19], yet more likely to reside with the child, therefore this could be an underestimate of the true number. The estimated number of children living with adults with alcohol problems can be sourced to a 1998, EuroCare document [20]. This document indicates that the estimate is an extrapolation of data from Denmark and Finland, each using a different methodology. The same document reports UK alcohol consumption to be significantly less than most other EU countries, yet recent trends in the use and misuse of alcohol are contradictory [21]. It is unclear how alcohol problems were defined and if they relate to the UK definitions of misuse. It appears to reflect drinking at a level considered in the UK as hazardous in one of the surveys. Thus, the existing estimates used to inform current UK policy and setting of targets for the next decade are dated, not based on local epidemiological data sources and need improving and broadening to include the combination of alcohol and other co-existing problems that can lead to adversity.

In contrast to considerable policy investment in addressing the needs of children living with substance misusers and in identifying good practice, the underlying epidemiological evidence has fallen short. For policy and commissioning responses to adapt to meet the needs of both parental substance misusers and their children, we first need to understand the nature and scale of the problem. Without knowing the number of potentially at-risk families, we are unable to assist them until they come to the attention of agencies at crisis point. The current study set out to update, improve and broaden earlier estimates to include alcohol, drugs and multiple/elevated risk factors of harm e.g. concurrent mental distress and substance use. This was achieved through secondary analysis of existing national household surveys which have captured relevant data. Attempts to generate new data to answer this research question are likely to be hampered by social desirability effects, thus generating unreliable estimates. The study reports these new estimates relating to the number of children living with alcohol and drug misusing parents.

## Methods

The method used was developed by one of the authors (DB) in a similar project undertaken for the Australian National Commission on Drugs [22]. A literature search identified peer-review, grey literature and key government documents on children of substance misusing parents, but the focus of the research was on identifying national databases. Household, cohort or other large-scale surveys were considered if they met the following inclusion criteria; contained information on i) domestic arrangements, ii) adult substance use and iii) number of children in the household under the age of 16 years. Permission was sought for access to the original datasets from the corresponding departments/organisations and for the data to undergo secondary analysis to address the research questions. Once granted, study questionnaires were examined to identify potentially relevant variables. Since the surveys were not designed to address this research question but were undertaken by different organisations, with different objectives, at different points in time and using different assessment tools, wide variations in the estimated methods were anticipated. The databases were examined to identify the most common and robust indicators of substance use that could be applied. Variables were created or transformed to generate consistent variables across the datasets e.g. converting daily units of alcohol into weekly units, individual illicit drugs into drug classes and continuous variables (e.g. units consumed per week into categorical data e.g. hazardous drinking). Databases containing inconsistent or incompatible variables or with large amounts of missing data on variables of interest were excluded. Surviving variables were cleaned, edited and appropriate methods for the handling of missing data were applied. Anyone under the age of 16 was considered to be a child and only those living in the same household as adult substance users constituted a case. The number of adults (>15 years) fulfilling criteria for substance use/misuse was calculated initially, followed by the presence of any children in the same household and finally the number of those children. Measures were taken to ensure that when surveys contained more than one respondent per household (e.g. both parents) each child was only counted once. The number of children reported by the adult respondents to be living in the household formed the denominator (i.e. total number of children living with the sample) and not the number of child respondents, as these could be limited to only two children per household in some surveys. Once the number of children living with parental substance misusers was calculated (as a proportion of all children living with the respondents), the figures were extrapolated to the number of under 16's in the general population at that time and in the relevant countries (e.g. England, Scotland or UK), using the Office of National Statistics interactive population pyramid . All estimates were added to a summary table and confidence intervals calculated. Regular comparisons were made across datasets and revisions made to ensure that the most consistent indicators of substance use were applied.

The five national surveys accessed and providing appropriate data were; the General Household Survey (GHS), 2004; the Household Survey for England, (HSfE) 2004; the National Psychiatric Morbidity Study (NPMS), 2000; the British Crime Survey (BCS), 2004/5 and the Scottish Crime Survey (SCS) 2000. The GHS and HSfE household surveys were conducted around the same time and used the same measures of alcohol consumption (including indicators of binge drinking), although weekly consumption could only be calculated for a sub-sample (those reporting that they drink the same amount each day). Respondents had been asked "which day in the last week did you drink the most?" and were then asked to list how many of each type of alcoholic beverage they had consumed on this day. Each recorded beverage was converted into units of alcohol and summed to provide total units consumed on that day. The UK Government definition of binge drinking was calculated for the sample [18], i.e. 6 or more units in a single drinking occasion for women and 8 or more units for men. This is above (twice) the maximum recommended daily benchmark, stating that 'regular consumption of 2-3 units a day for women and 3-4 units a day for men does not lead to significant health risk'. We adopted the governments' definition of binge drinking as an accepted UK convention - this is not to imply that there is parental risk for all drinkers meeting these criteria, nor, indeed that there is no substance-related parenting risk in those who do not reach these thresholds. The NPMS contained data on problematic (hazardous, harmful and dependent drinking). *Hazardous drinking *(a pattern of alcohol consumption that increases the risk of harmful consequences for the user or others) was defined as a score on the Alcohol Use disorders Identification Test [23] of 8 or more. *Harmful drinking *(consumption that results in consequences to physical and mental health) was defined as a score of 16 or more. The Severity of Alcohol Dependence Questionnaire [24] was used to identify alcohol dependence in this survey. The two crime surveys and the NPMS were used to examine illicit drug use, the NPMS to look specifically at cumulative risks and the SCS to look at examples of harm resulting from substance misuse in the household.

## Results

### Parental Alcohol Misuse

Striking similarities were observed across the surveys in the rates of parenting (both around 30%) and drinking behaviour (see Table 1). Around 81% of the population were current drinkers and around 17% had engaged in binge drinking at least once in the week before interview. Consistent rates emerged indicating that around 34% of binge drinkers had at least one child in the household. These figures were extrapolated to the population in England (for the HSfE) or UK (for the GHS) in Table 2. Estimates from the two datasets were combined to generate a single estimate which was extrapolated to the UK population.

**Table 1:** Key findings relating to parental alcohol misuse from two 2004 national household surveys (HfSE, GHS).

**Survey**	**HSfE**	**GHS**
Year	2004	2004

Sample	6,704 adults	16,715 adults

% with at least 1 child <16 in the household	30.4%	29.0%

No. of children living in the household	1990	4163

Mean age of children <16	7.7 years	7.7 years

Current drinkers	82.1%	81.4%

Binge drinkers	16.7%	17.3%

Mental Health problem^1^	12.1%	2.5%

Binge drinker with 1+ child	35.3%	33.0%

Sub-sample ^2^		

Hazardous or Dangerous drinkers	5.1%	4.9%

Dangerous drinkers	1%	1%

Hazardous/Dangerous drinkers with 1+child	22.%	23.4%

Dangerous drinker with 1+child	20.0%	24.8%

^1^Defined by a score of 4+ on the GHQ in the HSfE and the presence of at least one ICD mental health problem in the GHS

^2 ^For a sub-sample - those reporting alcohol units consumed on heaviest day in the past week, number of drinking days in past week and reporting that they drink the same amount each day, weekly units could be calculated. Hazardous refers to those exceeding 14 and 21 units weekly for females/males respectively. Dangerous drinking refers to those exceeding 35 and 50 units weekly for females/males respectively. However this sample was too small to generate population estimates from these surveys.

**Table 2:** Estimates of children living in households with alcohol misusing adults based on table 1

**Category: living with**	**%**	**CI** **Lower**	**CI** **Upper**	**Estimate**	**Lower** **Estimate**	**Upper** **Estimate**
**GHS**						

at least one binge drinker (BD)	29.7	28.3	31.1	3,458,654	3,297,010	3,620,298

a BD who is the only adult in household	4.2	3.6	4.8	489,103	418,143	560,062

at least 2 binge drinkers	7.4	6.6	8.2	861,752	769,149	954,355

an adult with a mental health problem	2.5	2.0	3.0	291,133	235,902	346,363

a BD with a mental health problem	0.6	0.4	0.8	69,872	42,552	97,191

**HSfE**						

at least one binge drinker (BD)	27.8	25.8	29.8	3,237,393	3,008,163	3,466,623

a BD who is the only adult in household	3.4	2.6	4.2	395,940	303,213	488,668

at least 2 binge drinkers	9.9	8.6	11.2	1,152,885	1,000,072	1,305,698

an adult with a mental health problem	18.9	17.2	20.6	2,200,962	2,000,643	2,401,280

a BD with a mental health problem	4.5	3.6	5.4	524,039	417,970	630,107

**COMBINED GHS and HfSE**						

at least one binge drinker (BD)	29.1	28.0	30.2	3,388,782	3,256,612	3,520,952

a BD who is the only adult in household	3.9	3.4	4.4	458,014	401,454	514,575

at least 2 binge drinkers	8.2	7.5	8.9	957,666	877,727	1,037,606

an adult with a mental health problem	7.8	7.1	8.5	910,351	832,239	988,463

a BD with a mental health problem	1.9	1.6	2.2	221,437	181,695	261,178

Across the two surveys, 27.8 - 29.7% of children, representing a combined estimate of 3,388,782 (95% confidence interval 3,256,612 - 3,520,952) children in the UK aged under-16, lived with an adult binge drinker, 3.4 - 4.2% of children (representing a combined estimate of 458,014) lived in a household where the only adult was a binge drinker and 7.7 - 9.9% (representing a combined estimate of 957,666) lived with at least two binge drinkers (typically both parents). The combined datasets indicated that 1.9% (representing 221,437 children) lived with an adult binge drinker with concomitant psychological distress which may be exacerbated by their drinking behaviour. According to the NPMS 22.1% (representing 2,643,049) children lived with a hazardous drinker, 2.5% (298,988) with a harmful drinker and 3.7% (442,502) in households where the only adult was 'at least' a hazardous drinker. Respondents scoring more than 10 on the AUDIT then completed the SADQ, 5.9% (representing 705,611) of children lived with the 7% of drinkers who met criteria for (at least) mild alcohol dependence (see Table 3).

**Table 3:** Percentage of children living in households with substance using parents from the BCS, SCS and NPMS

**Survey**	**BCS**	**SCS**	**NPMS**
Year	2004/5	2000	2000

Sample (adults)	5604^1^	2998	8580

% with at least 1 child <16 in the household	41.1%	38.1%	31.0%

No. of children living in the household	1975	2006	4783

Mean age of children <16	7.7	7.6	-

% children living with:			

an adult drug user (past year)	8.4%	4.9%	8.0%

an adult drug user (past month)	4.2%	3.3%	3.9%

an adult using class A drug (past year)	2.2%	1.4%	1.8%

an adult using class B drug (past year)	1.4%	0.4%	1.4%

an adult using class C drug (past year)	7.4%	4.2%	7.3%

an adult using class A drug (past month)	1.0%	0.9%	0.6%

an adult using class B drug (past month)	0.3%	0.2%	0.5%

an adult using class C drug(past month)	3.9%	2.7%	3.8%

a daily drug user	0.6%	0.8%	1.1%

only a drug using parent (past year)	5.7%	-	2.3%

only a drug using parent (past month)	3.3%	-	1.3%

an adult who has injected a drug	-	0.3%	0.6%

an adult who has overdosed from drugs	-	-	0.9%

an adult who is drug dependent	-	-	2.8%

an adult in drug treatment	-	-	0.6%

an adult who is a hazardous drinker (>8 on AUDIT)	-	-	22.1%

an adult who is a harmful drinker (>16 on AUDIT)	-	-	2.5%

an adult who is a dependent drinker (SADQ)	-	-	5.9%

only a hazardous or worse drinker	-	-	3.7%

^1^5604 answered the drugs section of the interview but total sample was 51,964

### Parental Drug Use

The proportion of children living with illicit drugs users was relatively consistent across the two UK surveys, albeit slightly lower in the SCS (See Table 3 &4). It was not possible to collapse the datasets to form a single estimate because they were conducted in different years. According to the BCS, 8.4% (representing 978,205 children) in 2004/5 (95% CI 835,739 - 1,120,671) lived with an adult who had used illicit drugs in the past year. A similar estimate of 8.0% (representing 956,760 children) in 2000 (95% CI 864,809 - 1,048,711) emerged from the NPMS. The figure for Scotland in 2000 based on the SCS was 4.9% (representing 47,631 children). The UK surveys suggest that 3.9% of children in the year 2000 and 4.2% of children in 2004/5 lived with an adult who had used illicit drugs in the previous month (representing around 466,420 and 489,103 respectively). 1.8% of children (in 2000) and in 2.2% of children (in 2004/5) lived with a class A drug user (representing 215,271 and 256,197 respectively), whilst 0.6% and 1% respectively lived with someone who had used a class A drug in the previous month. 7.3% (in 2000) and 7.4% (in 2004/5) (representing 873,043 and 861,752 respectively) lived with a class C drug user, 3.8% and 3.9% respectively, with someone who had done used a class C drug in the previous month. Living in a household where the only adult was a drug user was the case for 2.3%, representing 275,069 children in 2000 and 5.7%, representing 663,782 children in 2004/5. The rates are higher for class C than class B drugs because cannabis was classified as a class C drug at the time analysis was conducted.

**Table 4:** Estimates of children living in households with substance using adults based on table 3

**Children living with an adult who has/is......**	**%**	**CI** **Lower**	**CI** **Upper**	**Estimate**	**Lower** **Estimate**	**Upper** **Estimate**
**BCS**						

used drugs in past year	8.4	7.2	9.6	978,205	835,739	1,120,671

used drugs in past month	4.2	3.3	5.1	489,103	386,080	592,125

used drugs class A past year	2.2	1.6	2.8	256,197	180,860	331,533

used drugs class B past year	1.4	0.9	1.9	163,034	102,691	223,377

used drugs class C past year	7.4	6.2	8.6	861,752	727,307	996,197

used drugs class A past month	1.0	0.6	1.4	116,453	65,351	167,555

used drugs class C past month	3.9	3.0	4.8	454,167	354,737	553,597

a daily drug user	0.6	0.3	0.9	69,872	30,208	109,535

used drugs in past year and is a lone parent	5.7	4.7	6.7	663,782	544,708	782,856

**SCS**						

used drugs in past year	4.9	4.0	5.8	47,631	38,448	56,814

used drugs in past month	3.3	2.5	4.1	32,078	24,479	39,677

used drugs class A past year	1.4	0.9	1.9	13,609	8,611	18,607

used drugs class B past year	0.4	0.1	0.7	3,888	1,203	6,573

used drugs class C past year	4.2	3.3	5.1	40,827	32,294	49,360

used drugs class A past month	0.9	0.5	1.3	8,749	4,731	12,766

used drugs class C past month	2.7	2.0	3.4	26,246	19,351	33,141

a daily drug user	0.8	0.4	1.2	7,777	3,987	11,566

an injecting drug user	0.3	0.06	0.5	2,916	590	5,243

**NPMS**						

used drugs in past year	8.0	7.2	8.8	956,760	864,809	1,048,711

used drugs in past month	3.9	3.4	4.5	466,420	400,804	532,0376

used drugs class A past year	1.8	1.4	2.2	215,271	170,209	260,333

used drugs class B past year	1.4	1.1	1.7	167,433	127,611	207,255

used drugs class C past year	7.3	6.6	8.0	873,043	784,874	961,213

used drugs class A past month	0.6	0.4	0.8	71,757	455,82	97,932

used drugs class C past month	3.8	3.3	4.3	454,461	389,658	519,264

a daily drug user	1.1	0.8	1.4	131,555	96,203	166,906

an injecting drug user	0.6	0.4	0.8	71,757	45,582	97,932

overdosed	0.9	0.6	1.2	107,636	75,626	139,645

drug dependent	2.8	2.3	3.3	334,866	278,951	390,781

in drug treatment	0.6	0.4	0.8	71,757	45,582	97,932

used drugs in past year and is a lone parent	2.3	1.9	2.7	275,069	224,261	325,876

Hazardous drinker (AUDIT)	22.1	20.9	23.3	2,643,049	2,502,418	2,783,681

Harmful drinker (AUDIT)	2.5	2.1	2.9	298,988	246,071	351,904

Dependent drinker (SADQ)	5.9	5.2	6.6	705,611	625,749	785,472

Only adult is at least a hazardous drinker	3.7	3.17	4.2	442,502	378,523	506,480

According to the SCS, 0.3% (representing 2,916 children in Scotland) and according to the NPMS 0.6% (representing 71,757 children in the UK) were living with an injecting drug user. The NPMS indicated that 0.6% (71,757 children) lived with a drug user in treatment, 2.8% (334,866) lived with a drug dependent user, 0.9% (107,636) lived with an adult drug user who had experienced a drug overdose and 1.1% (131,555) with a daily drug user. The number of children living with a lone drug using parent in 2004/5 had doubled at 5.7% (representing 663,782 children) from the estimated 2.3% (representing 275,069) in the UK in 2000.

### Cumulative and more severe risks of harm

**Figure 1:**
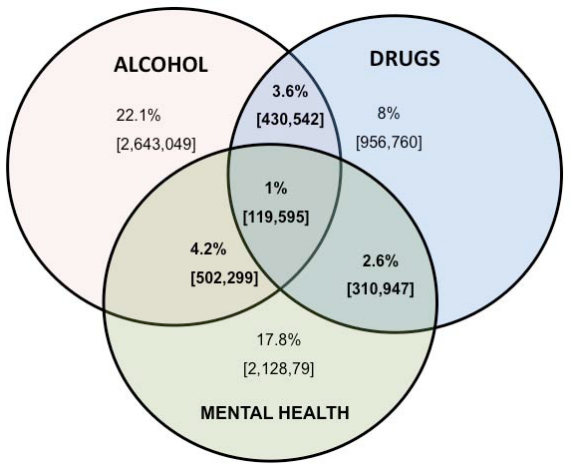
**Cumulative risk of harm from the NPMS**.

The NPMS contained information on drinking (using standardised assessment tools such as SADQ and the AUDIT), illicit drugs and mental health status using a standardised psychiatric assessment tool (CIS-R). This enabled an estimate to be generated for the number of children exposed to multiple or cumulative risk (see Table 5 and Figure 1).

**Table 5:** Population estimates of children living with adults where there is cumulative risk (NPMS)

**Children living with an adult who is a...**	**%**	**CI** **Lower**	**CI** **Upper**	**Estimate**	**Lower** **Estimate**	**Upper** **Estimate**
problem drinker & drug user (past year)	3.6	3.1	4.1	430,542	367,402	493,683

problem drinker & drug user (past month)	1.8	1.4	2.2	215,271	170,209	260,333

problem drinker with mental health problems	4.2	3.6	4.8	502,299	434,312	570,286

drug use (past year) with mental health problems	2.6	2.2	3.1	310,947	257,010	364,884

problem drinker, drug user (past year) + mental health problems (past year)	1.0	0.7	1.3	119,595	85,871	153,319

problem drinker, drug user (past year) + mental health problems (past month)	0.6	0.4	0.8	71,757	45,582	97,932

Three point six percent of children (430,542) in the UK in 2000 lived with an adult problem drinker (a score of more than 8 on the AUDIT) who had used drugs in the previous year, and 1.8% (215,271) with a problem drinker who had used drugs in the previous month. Around 4.2% (502,299) lived with a problem drinker with a concurrent mental health problem (a score of >12 on CIS-R), and 2.6% (310,947) lived with a drug user with a concurrent mental health problem. Even greater risk of harm could have existed for the 1% (119,595) of children who lived with an adult problem drinker who also used drugs and had concurrent mental health problems.

Finally, the SCS contained examples of circumstances where harm can result directly from drug or alcohol misuse (see Table 6). It recorded incidents when violence (use of force, push/shove, thrown, threatened, chocked/strangled, hit, stabbed, forced sex and other) was used on an adult (usually a parent) in the house by another adult (usually their partner) after drinking/drug use, and when these acts of violence were witnessed by children in the household. This indicated that in Scotland, 2.5%, (24,302 children) lived in a household where violent incidents occurred after the perpetrator had been drinking and 1.2% (11,665) lived in a household where violent incidents occurred after the perpetrator had been using drugs. Even greater harm may exist for the 1.2% (111,665) and 0.6% (5832) who witnessed these acts of violence occurring as a result of alcohol and drug use respectively.

**Table 6:** Population estimates of children living in households where violence occurs following substance use (Scottish Crime Survey, 2000)

**Children living in a household where there is...**	**%**	**CI** **Lower**	**CI** **Upper**	**Estimate**	**Lower** **Estimate**	**Upper** **Estimate**
violence in household when adult was drinking	2.5	1.8	3.2	24,302	17,660	30,943

violence in household when adult was using drugs	1.2	0.7	1.7	11,665	7,033	16,297

violence in household witnessed by child when adult was drinking	1.2	0.7	1.7	11,665	7,033	16,297

violence in household witnessed by child when adult was using drugs	0.6	0.3	0.9	5,832	2,547	9,118

## Discussion

Overall, the figures suggest that the number of children living with at least episodic binge drinkers or illicit drug users is greater than previously thought. In 2004, 3.3 - 3.5 million children in the UK were living with at least one binge drinking parent. Having a non-binge drinking adult in the household offers a positive role model and can mitigate against harm caused by the problem drinking parent [25]. Therefore the near half a million children living with a lone-binge drinking parent and 957,000 with two binge-drinking parents could be more vulnerable to harm. Whilst there is no evidence to suggest that parental binge drinking is associated with harm to children, adults in this category were 'at least' binge drinking. Some would have been problem drinkers and there is literature emerging to suggest that problem drinking is associated with childhood adversity [14-16]. The estimated 2.6 million living with a hazardous drinker in the year 2000 would appear to exceed the earlier estimates of 780,000 - 1.3 million, although it is not clear how problem drinking was defined in the estimates from data sources in Denmark and Finland. Whilst the data does not imply that these children experience adverse consequences, the potential for exposure (assuming it occurs in the home) to modelling heavy drinking behaviour exists, as does neglect and less adequate parental responses to accidents and emergencies (child injuries, fires and other adverse events which are more likely to occur in the event of intoxicating substance use). These new estimates complement the existing estimates on treated addiction populations and add to what we know. Unfortunately, however they remain a long way from what we need to know.

Around one million children in the UK live with an adult who has used an illicit drug in the past year, and just under half a million with someone who has done so in the past month. It is not possible to compare directly with the Hidden Harm (2003) estimates since they are generated from different populations and using different methodologies. It is plausible that illicit drug use could constitute smoking cannabis when the drug user does not have responsibility for child care, thus posing no acute risk of harm. Although it could be argued that any drugs use can create a social learning model and that regular use may result in chronic effects that are more likely to compromise parenting capabilities. Equally, however it could constitute regular use of cocaine or heroin in the home environment, where the child could be exposed to drug taking behaviours, paraphernalia, dealers, and the potential to ingest or experiment with the drug. The finding that the number of children living in a household where the only adult was a drug user had more than doubled between 2000 and 2004/5, points to increasing vulnerability in single-parent families and highlights the need for child protection efforts to determine need, as well as risk. The finding that 334,000 children were estimated to be living with a dependent drug user is broadly consistent with the Hidden Harm (2003) estimate relating to treated drug users. The finding that 107,000 children lived with an adult who had experienced a drug overdose, is an indicator of the possible severity of drug misuse among this predominantly untreated population. Given that it is estimated that there are 116,809 injecting heroin or crack cocaine users in England alone in 2006/7 [26], the current estimate of only 72,000 children living with an injecting drug user in the UK is low and may reflect a reluctance to disclose injecting behaviour in the context of household surveys.

The potential for cumulative disadvantage for children living with adults with multiple problem behaviours is a particular concern as co-morbidity has been linked to less effective treatment engagement and additional difficulties in parenting [27,28]. Parental mental illness featured in one-third of 100 reviews of child deaths of abuse and neglect[29]. Parental substance misuse was a concern for 52% of families placed on child protection registers and for 62% of children subject to childcare proceedings [30,31]. Therefore the risk of harm to children of parents with co-morbid substance misuse and mental health problems is likely to be even greater. Parental experience of blunted emotions/feelings, anxiety or depression in addition to substance use may restrict the child's social and recreational activities. Finally, the observation that large numbers of children have witnessed violence occurring in the context of substance misuse is a major concern for child protection agencies and supports earlier findings [32,33].

There are some limitations with this work that are worthy of consideration. It is important to recognise that the new estimates are likely to be conservative estimates and subjected to measurement and reporting bias. Significant underreporting of substance use is likely to have occurred, given that data were gathered in the respondents home, provided by parents and with questions relating to child welfare. Extrapolation of survey data to the UK population, poses the risk of accentuating sampling or response biases. However, the concordance of several estimates across surveys mitigate this concern to some degree. Nonetheless we recognise that there are intrinsic limitations to population surveys that make extrapolations to populations risky, and these include the under-representation of certain ethnic groups, the homeless and other vulnerable populations and the over-representation of more stable groups. The disparate rates of psychological distress reported in the GHS and HSfE are almost certainly an artefact of the different assessment tools used (GHQ-12 versus ICD-10 codes). Future research should aim to generate estimates based around different ages, since the risk and types of harms to babies and young children are likely to differ from those of teenagers. A limitation of household surveys is that they are cross-sectional in nature with respondent substance use and parental status measured at a single point in time, yet these are not stable factors but fluctuate over time. It may be more helpful to generate estimates using databases with multiple and regular assessment intervals e.g. longitudinal or cohort studies such as the Millennium Cohort Study to identify changes in exposure to risk due to factors such as child age.

The findings should be used to inform the design and content of future research aiming to explore the ways in which parenting capacity is affected by their substance use. Despite a better indication of the scale of the problem, the absence of contextual data limits the conclusions that can be drawn for both policy and practice. However, the relatively uncritical citing of the estimates used in the current UK drug and alcohol strategies should be challenged. Future research needs to examine the relative risks of harm posed to children with different substances used and patterns of use i.e. chronic patterns of relatively low-level substance use as well as those associated with binge or intoxicating use of substances. Survey items designed to capture the nature of harm must be discretely embedded within a broader research interview to prevent social desirability effects. The use of multiple methodologies will overcome the reliance on secondary analysis of surveys designed for other purposes.

While we must be cautious about the historical and opportunistic nature of the data presented here, these findings have implications for policy and practice, concerning the early identification of parental substance misuse and provision of early interventions. These can serve to minimise adverse childhood experiences and halt the escalation of recreational/binge substance use to that of problematic/dependent use more likely to cause harm. Possible indirect pathways of harm include unemployment, physical, psychological, social and financial substance-related problems and stigma [34], all of which can impede child development [1]. Whilst the bulk of the data presented here captures binge drinking and recreational drug use, universal and generic services working with parents and children should attempt to assess and be sensitive to parental substance use and possible related adversity that can serve as markers of harm. It may be time to revise the questions routinely asked when assessing parenting capacity in the Common Assessment Framework (CAF) and through Local Children's Safeguarding boards.

Research conducted on the caseloads of social services and other child welfare agencies [31,35] highlight the information, assessments and interventions required to manage risk where parental substance use exists. However, there are also implications from these findings for agencies that aim to encourage the uptake of substance use treatment and for universal educational initiatives aiming to raise awareness of the harms parental substance use can pose to their children. Training in effective management of the daily stressors associated with socioeconomic disadvantage, improving parenting practices by providing a supporting and nurturing environment for a child, and educating women about drinking during pregnancy may halt the progression from risk to actual harm.

Finally, it must be borne in mind that whilst the number of children living with parental substance misusers appears greater than earlier estimates, the picture is not universally bleak. Research findings indicate that the most high-risk drug taking behaviours tend to exist among parental substance misusers whose children live elsewhere, thus eliminating direct negative effects [36,37]. Whilst substance misuse can impair parenting capacity, harm is not inevitable and rarely exists as a consequence of substance use in isolation. Poverty, social exclusion, poor housing, a stressful environment, family tension and conflict and lack of psychosocial resources etc. collectively heighten the risk of harm [9]. This makes effective inter-agency working, involving the sharing of responsibilities, information on the potential indicators of harm and comprehensive assessments of need, harm and risk a top priority. Generating local evidence bases around this topic is as important as structured research. The 2006 'Working Together to Safeguard Children Report' [11] states that 'it is the duty of all agencies working with parents and children to make arrangements to safeguard and promote the welfare of the child', and places a heavy emphasis on sharing and effective joint working. Further recommendations are made in the recent 'The Protection of Children in England: A Progress Report' [38], including a need to develop guidance on referral and assessment systems for children affected by parental substance misuse, using current best practice. Many substance users, particularly the stable ones in treatment, can be competent and loving parents. Nonetheless, it is important that efforts are not focussed solely on these most vulnerable families and to recognise that widespread patterns of binge drinking and recreational drug use may also disadvantage children and expose them to a poorer standard of care and safety than is acceptable.

## Conclusion

Estimating the number of children living with parental substance misusers and in particular those who warrant professional intervention poses several challenges. Characterised by denial and shame, the numbers can only be inferred from available data provided by parents and the reliability of such self-report data is unknown. Nonetheless, secondary analysis of the most recent household surveys in the UK indicates that taken together, the number of children living with substance users and binge drinkers is far greater than earlier estimates would suggest. The US and emerging UK literature suggests that interventions delivered within social services and substance misuse agencies, incorporating family therapy and aiming to improve parenting skills, show much promise for families where harm already exists. However, it is the majority of those children raised in households where one or both parents are problem substance users without professional or mainstream services intervention who may experience the greatest adversity. Whilst actual harm from parental substance misuse is not inevitable, only large scale and far-reaching initiatives will likely impact on the 3.4 million children living with binge drinkers and almost 1 million living with drug users, where the potential for harm exists. In light of new findings on the scale of the problem, we urge mainstream services to reflect on their role in supporting these vulnerable families and in raising awareness of how parental substance use can elevate the risk of harm to children. Improving access to treatment, family interventions and parenting skills training, should place us in a stronger position than ever to prevent harm to children of substance-misusing parents.

## Competing interests

The authors declare that they have no competing interests.

## Authors' contributions

VM was responsible for the study design, data collection and analysis and writing the manuscript. DB was responsible for study design, analysis, development of methodology and assisting with the writing of the manuscript. Both NF and ET were responsible for identifying data sources, data collection and analysis and assisting with manuscript revisions. All authors read and approved the final manuscript.

## Pre-publication history

The pre-publication history for this paper can be accessed here:


